# Progress in Psychiatry

**Published:** 1902-01-25

**Authors:** 


### PROGRESS IN PSYCHIATRY.

(*Continued from page* 272.)

**Pathogenesis of Melancholia with Delusions.**—In considering the pathogenesis the following facts regarding the disease must be constantly kept in mind. First that stresses which normally produce reactions of pain and fear in the healthy and robust may in the predisposed and in anxious and timid persons produce melancholia, *i.e.*, reactions out of all proportion to the stress or stimulus. Secondly, that the emotional predominance of such states of fear and pain imply corresponding disturbances of cerebral and bodily functions, and therefore that persons prone to such cerebral and bodily disturbances (from whatsoever causes proceeding) are, *ipso facto*, also prone to melancholia. (The cerebral and bodily conditions in question include tendencies to dyspepsia, "biliousness," and constipation—common especially in women—insomnia and cephalagia from toxæmias, brought about by the accumulation in the system of nitrogenous bodies of the purin group, and defectiveness in the excretory powers of the kidneys and skin.) Thirdly, that a high degree of psychical development as regards intelligence and emotional sensibility is necessary for the development of melancholia in its most marked forms. A diminution or loss of the normal feeling of well-being marks the *first grade* in melancholia. There is a slight degree of listlessness and depression such as may be produced by fatigue, by partial insomnia or inanition, or as the result of mild toxæmia (alcoholic), or of sexual excesses. Mental lucidity is preserved and the condition is one capable of rapid restoration to good health. The *next grade* is marked by a persistence and a predominance of fears and doubts (obsessious) affecting the *ego* at first vaguely and indefinitely, but compelling its attention thereto and thus leading to moods of brooding and self-absorption. The kinæsthetic activities are thus diminished, and the melancholiac shrinks into retirement and solitude just as a wounded animal would do. But unlike the animal, the melancholiac with his larger and more complex psychical life is not thus able to escape from its anxieties and responsibilities, fears, doubts, and painful obsessions continue to occupy his mind, he suffers from sleeplessness and loss of appetite, and symptoms of gastro-intestinal disorder and of toxæmia show themselves. A vicious circle is thus established, and the morbid processes in brain, bowel and blood tend to be perpetuated until exhaustion occurs in the nervous centres concerned. The *third grade* is thus produced. Mental lucidity is clouded and obscured at this stage, and attacks of terror and of agitation (panophobic crises) may be manifested at intervals. The physical symptoms accompanying this stage are constipation, albuminuria and ischuria,[7] slight febrile temperature, total insomnia, contracted arterioles with a high radial blood-pressure, arrest of perspiration, muscular weakness, and myotatic irritability. The gestures and acts may be expressive of agitation (melancholia agitans), or the attitude may be one of stupor (m. attonita) or a combination of poses of fear and repugnance maintained with some degree of plasticity (m. catatonica[8]), and punctuated by meaningless phrases and sounds (verbigeration). The logical reasoning processes are decidedly weakened at the close of the second stage, and the recurrence of painful obsessions during the third stage tends further to disturb the mental balance. Hallucinations of hearing and of other senses, and illusions of taste and of bodily feeling become prominent during the third stage. The association-centres of the brain (sphere of intelligence) are weakened and disturbed in function by the recurrent obsessions, hallucinations and illusions which invade them and which thus influence conduct. Delusions are thus originated, for such delusions are nothing more than the notions and beliefs aroused in the sphere of intelligence by the invading obsessions, hallucinations and illusions, and outwardly expressed by speech, gesture, or conduct. Delusions may be of different kinds, and may have all degrees of persistency and power to influence conduct. The delusions of the melancholiac are always of a painful nature, and expressive of suffering and fear, of hopelessness and despair. A feeling of powerlessness to combat or of hopelessness to overcome the malign aspects of the environment often leads the melancholiac to suicidal thoughts. Even in the milder grades of melancholia such thoughts are common, but the reasoning power still asserts itself, the sense of honour and of responsibility is still recognised, and the suicidal inclinations are counterbalanced or overcome by self-control. But the possibility of danger should be borne in mind, and mishaps guarded against by the constant presence of a nurse or attendant. *General prognosis.*—Simple melancholia may either end in recovery within a few days or weeks, or may gradually pass into a graver stage. In the latter case the patient becomes more depressed and self-absorbed, refuses food and medical treatment, and develops delusions. The stage of vague fear and apprehension has passed into one of confirmed despair and misery, and delusions which were at first occasional and fleeting now become powerful and persistent. Hardly any two patients present exactly the same delusions, and owing to their variety and number the delusions are hard to classify. They may, however, conveniently be grouped into two main classes, viz., hypochondriacal delusions relating to limited parts of the body, and delusions of pain and fear affecting the ego generally (so-called psychical delusions). Of the latter class, delusions of a religious character are both frequent and prominent, and have been already spoken of. Delusions of "voices" uttering insults and imprecations, or of people in the street mocking or abusing the patient are frequently entertained. Illusions of taste and smell probably arise from disordered secretion, and from such illusions the transition is easy to delusions of poisons being administered in the food or of poisonous vapours administered by enemies. In hypochondriacal melancholia the delusions refer solely to parts of the body, the stomach, bowels, lungs, heart, genitalia, limbs, hair and nails, skin, or other parts. Delusions of being infected with some loathsome disease, such as syphilis or cancer, of having gangrene or mortification of a limb, or of parasites on or in the body, complete the list of the hypochondriacal complaints. Many of these cases do actually have some organic trouble as the basis of their delusions, a condition of gastric catarrh, enteroptosis, a chronic bronchitis, floating kidney, or some simple skin affection or benign tumour. Patients of this class are pictures of abject misery, wholly absorbed in their own complaints, loud in complaining and in expatiating on their disorders, real or fanciful. Suicidal attempts are made by such patients, not necessarily to obtain relief from suffering, but more often to gain sympathy, attention, and notoriety. In alcoholic patients[9] symptoms of polyneuritis complicate melancholia and show themselves in the form of pricking and formications, numbness and tinglings of the extremities, darting pains and "electric" twitchings of the skin, and the appearance of areas of analgesia and hyperæsthesia of the skin. In agitated melancholia the delusions and mental suffering are manifested by incessant movement, such as tearing of the hair, wringing the hands, sobbing and weeping, striking of the forehead and breast with the hands, and repeated interjections and phrases of woe. Ideas of eternal damnation have a peculiar fascination for such patients, and as long as the state of agitation exists the patient remains thin and anæmic. When recovery or lapsing into dementia is occurring, the patient commences to get fat. In the stuporous (including the catatonic) forms of melancholia, the degree of mental alienation reached is very grave; the patient is mute and passive, entirely absorbed in and stupefied by his sufferings, absolutely fascinated with horrors, and apparently oblivious of what is taking place around. The capacity for action and volition seems to be totally arrested, and passive resistance is offered to everything which is done as regard nursing, feeding, and treatment. This form occurs in neuropathic subjects, often after a severe fright or shock, or serious illness. The stuporous condition is more apparent than real, as is shown by the anxious expression, the facial twitchings, and the occasional starts and frantic attempts at violence or self-destruction. After recovery a vague and confused memory, or total amnesia, of what occurred during the illness, may be noted. The muscular condition is one of slight contracture and inability to change from one attitude to another. The circulation is feeble, the extremities cold and blue, and nutrition very low. Occasionally the mutism may give place to verbigeration, and the fixity of attitude give place to poses expressive of fear and repugnance. The prognosis is grave.

7 Bruce, Journal of Mental Science, October, 1900. 8 Tuke's Dictionary of Psychological Medical, sub voce Katatonia; also Nolan in the Journal of Mental Science, 1892. 9 Berkley's Mental Diseases, 1901.

